# Risk Factors for Hepatitis C Infection Among Sexually Transmitted Disease-Infected, Inner City Obstetric Patients

**DOI:** 10.1080/10647440300025520

**Published:** 2003

**Authors:** Youyin Choy, Lisa Gittens-Williams, Joseph Apuzzio, Joan Skurnick, Carl Zollicoffer, Peter G. McGovern

**Affiliations:** 1 Division of Maternal-Fetal Medicine Department of Obstetrics Gynecology and Women’s Health UMDNJ-New Jersey Medical School, 185 South Orange Avenue, MSB-E506 Newark NJ 07103 USA; 2 Preventive Medicine and Community Health UMDNJ-New Jersey Medical School Newark NJ USA

## Abstract

*Objective:* To test the hypothesis that our inner city obstetric patients who have been infected with sexually transmitted diseases (STDs) will have a higher prevalence of hepatitis C virus infection than the general population and to identify specific risk factors and high-risk groups.

*Methods:* All patients in our prenatal clinic (July 1997–April 1999) who tested positive for one or more STDs were asked to return for hepatitis C antibody testing. Medical charts of all patients who returned for hepatitis C testing were reviewed.

*Results:* A total of 106 patients with STDs were tested for hepatitis C. Positive screening tests for anti-hepatitis C antibody were found in 6.6% (7/106) of the patients (95% CI = 2.7–13.1%). This frequency is significantly higher than the hepatitis C prevalence (1.8%) in the general United States population (*p* = 0.006). Multiple logistic regression analysis confirmed only older age (*p* = 0.016) and positive HIV status (*p* = 0.023) to be significant predictors of hepatitis C infection.

*Conclusions:*  Inner city STD-infected obstetric patients are at high risk for hepatitis C infection compared with the general population. Increasing age and HIV-positive status are risk factors which are significantly associated with hepatitis C infection.


Infect Dis Obstet Gynecol 2003;11:191–198

### Risk factors for hepatitis C infection among sexually transmitted disease-infected, inner city obstetric patients

**Youyin Choy[1], Lisa Gittens-Williams[1], Joseph Apuzzio[1], Joan Skurnick[2], Carl Zollicoffer[1] and Peter G. McGovern[1]**

[1]*Departments of Obstetrics, Gynecology and Women's Health, and*
[2]*Preventive Medicine and Community Health, UMDNJ-New Jersey Medical School, Newark, NJ*

***Objective:*** To test the hypothesis that our inner city obstetric patients who have been infected with sexually transmitted diseases (STDs) will have a higher prevalence of hepatitis C virus infection than the general population and to identify specific risk factors and high-risk groups.
***Methods:*** All patients in our prenatal clinic (July 1997–April 1999) who tested positive for one or more STDs were asked to return for hepatitis C antibody testing. Medical charts of all patients who returned for hepatitis C testing were reviewed.
***Results:*** A total of 106 patients with STDs were tested for hepatitis C. Positive screening tests for anti-hepatitis C antibody were found in 6.6% (7/106) of the patients (95% CI = 2.7–13.1%). This frequency is significantly higher than the hepatitis C prevalence (1.8%) in the general United States population ($p$ = 0.006). Multiple logistic regression analysis confirmed only older age ($p$ = 0.016) and positive HIV status ($p$ = 0.023) to be significant predictors of hepatitis C infection.
***Conclusions:*** Inner city STD-infected obstetric patients are at high risk for hepatitis C infection compared with the general population. Increasing age and HIV-positive status are risk factors which are significantly associated with hepatitis C infection.

Key words: HEPATITIS C; SEXUALLY TRANSMITTED DISEASE; PREGNANCY

Infection with the hepatitis C virus is the most common blood-borne infection in America. The prevalence in the general United States population is 1.8%. It is estimated that over 3.9 million Americans are currently infected and that 95% of these individuals are unaware of their infection[1]. About 75% of acute hepatitis C infections are asymptomatic; 80% of these patients will go on to have chronic infection[1]. Of patients with chronic infection, 25% will develop cirrhosis and 20% will progress to hepatocellular carcinoma in an average of 25 years[1]. Therefore, early identification of hepatitis C infection is important in developing programs for prevention and possible treatment with anti-viral therapy.

Universal screening for hepatitis C infection has not yet been recommended due to the low prevalence rate in the general population. The Centers for Disease Control and Prevention[1] (CDC) have recommended that hepatitis C screening should be offered to patients at high risk for infection. Routine screening for hepatitis C infection is recommended for the following high-risk groups: patients with a history of injecting drug use, patients who received blood transfusion or organ transplants before 1992, patients

Correspondence to: Lisa Gittens-Williams, MD, Division of Maternal-Fetal Medicine, Department of Obstetrics, Gynecology and Women's Health, UMDNJ-New Jersey Medical School, 185 South Orange Avenue, MSB-E506, Newark, NJ 07103, USA. Email: gittenln@umdnj.edu

© 2003 The Parthenon Publishing Group who received clotting factor concentrates before 1987, patients with a history of chronic hemodialysis or chronic liver disease, and children born to hepatitis C-infected women. Hepatitis C is primarily transmitted via percutaneous exposure – the most commonly reported risk factor is the use of parenteral drugs (60%). Blood transfusion (which accounted for the majority of hepatitis C infections acquired more than 10 years ago) is currently responsible for only a small percentage of cases.

Sexual transmission of hepatitis C seems to occur[1–3]. Data are still lacking, however, regarding the extent to which sexual activity and STDs contribute to the transmission of the hepatitis C virus[1]. Intranasal drug use has also been reported as a risk factor for hepatitis C infection[4]. Due to the lack of consistent data, the CDC have determined that hepatitis C screening is of 'uncertain' need for those with a history of multiple sexual partners, a history of STDs or intranasal cocaine or other non-injecting drug use.

Both STDs and substance abuse are quite common among our inner city population. We undertook this study to test the hypothesis that hepatitis C infection would be more common in our inner city STD-infected pregnant patients than in the general United States population. Our goals were: (i) to define the prevalence of hepatitis C in our STD-infected pregnant patients and (ii) to identify specific high risk groups in which screening for hepatitis C would be most productive.

### SUBJECTS AND METHODS

All pregnant women in our clinic undergo routine testing for STDs as part of routine prenatal care. This testing includes cervical swabs for chlamydia and gonorrhea and serum tests for syphilis, hepatitis B, and HIV. With the prior approval of our study protocol by our Institutional Review Board, all pregnant patients receiving care at University Women's Health Center, New Jersey Medical School, Newark, from July 1997 to April 1999 who tested positive for one or more STDs during prenatal visits were asked to return for hepatitis C screening. Criteria for STDs were defined as positive tests for: chlamydia by polymerase chain reaction (PCR), gonorrhea by PCR or Thayer–Martin agar culture (as required by insurance carrier), syphilis by VDRL confirmed by fluorescent treponeme antibody, hepatitis B by B surface antigen or B core antibody or HIV by enzyme-linked immunosorbent assay (ELISA) confirmed with Western blot. All patients who returned for the requested hepatitis C screening test were included in our analysis.

The anti-hepatitis C antibody screening test used by our laboratory was an enzyme immunoassay (hepatitis C virus encoded antigen–recombinant c100-3, HC-31 and HC-34, Abbott hepatitis C EIA 2.0, 1992, Abbott Laboratories, Abbott Park, IL). Patients with known hepatitis C infection were excluded from the study. If the screening hepatitis C antibody test was positive, then the patients were asked to return for confirmatory testing with a recombinant immunoblot assay (hepatitis C virus encoded antigen–recombinant 5–1–1, c100–3, c33c and c22–3, Chiron RIBA hepatitis C 2.0 SIA, 1995, Chiron Corporation, Emeryville, CA).

We then reviewed the patients' charts and laboratory test results to collect information on STDs and demographic data including: age, gravidity, parity, number of sexual partners in the past 6 months and any history of drug use. Drug use was defined as chart documentation of a patient self-report of illicit drug use during an encounter with their health care provider. This was broken down into injection use (intravenous or skin-popping) or intranasal use.

Data analysis was performed using StatXact and LogXact statistical software. Confidence intervals for prevalence rates were based on the binomial distribution. The prevalences of various infections in different groups were compared using Fisher's Exact Test. The rank sum test was used to compare continuous and ordered variables (age, parity) of hepatitis C-infected women and hepatitis C-negative women. Multiple logistic regression was used to test the simultaneous significance of variables as independent risk factors for hepatitis C infection. The criterion for statistical significance was two-tailed $p < 0.05$.

### RESULTS

Table 1 presents the prevalence of various STDs in the population tested. A total of 106 STD-infected pregnant patients were screened for hepatitis C antibody and seven had a positive screening test, giving a prevalence of hepatitis C of 6.6%. This is significantly higher than the control general United States population with a reported 1.8% prevalence (95% CI = 2.7–13.1%, $p = 0.006$).

**Table 1** Prevalence of the different STDs in the study population

| *STD* | *Prevalence of STD (%)* | *STD-infected* | *Total number tested* |
|---|---|---|---|
| Chlamydia | 56 | 59 | 106 |
| Hepatitis B | 31 | 33 | 106 |
| Gonorrrhea | 16 | 17 | 106 |
| HIV | 6.9 | 7 | 102 |
| Hepatitis C | 6.6 | 7 | 106 |
| Syphilis | 3.8 | 4 | 106 |

**Table 2** Demographic characteristics of patients sero-positive for hepatitis C and patients who are seronegative

| *Population characteristic* | *Hepatitis C-positive median (95% CI)* | *Hepatitis C-negative median (95% CI)* | *Rank sum test p-value* |
|---|---|---|---|
| Age | 28 (21–45) | 21 (14–39) | – |
| | ($n = 7$) | ($n = 99$) | 0.001 |
| Gravidity | 3 (2–6) | 2 (1–11) | – |
| | ($n = 6$) | ($n = 94$) | 0.031 |
| Parity | 1.5 (0–3) | 0 (0–6) | – |
| | ($n = 6$) | ($n = 94$) | 0.17 |

Chlamydia was the most common STD in the studied population, identified in 56% of our STD-positive patients. This was followed in frequency by hepatitis B, gonorrhea and HIV infection. Hepatitis C infection was the fifth most common infection in this studied population. HIV infection was identified with almost the same frequency as hepatitis C (6.9 versus 6.6%). Syphilis testing had a much lower yield (3.8%) than hepatitis C screening.

Table 2 presents the population characteristics of our STD-positive pregnant patients who did or did not have hepatitis C infection. Patients testing positive for hepatitis C infection were significantly older (median age 28 versus 21 years, $p = 0.001$). Patient age alone identified a group of women at high risk for hepatitis C infection; of women less than or equal to 24 years, only 1/74 (1.35%) had hepatitis C, while 6/32 (18.75%) of those greater than or equal to 25 years were infected. They also had a significantly greater number of pregnancies than the hepatitis C-negative group (median gravidity of 3 versus 2, $p = 0.03$). Hepatitis C-positive women also tended to have more livebirths (median parity of 1.5 versus 0), although this trend did not reach statistical significance.

Table 3 presents the influence of various risk factors upon a patient's chance of testing positive for hepatitis C. Univariate analysis clearly demonstrated significant positive associations between hepatitis C infection and histories of intravenous drug use, nasal drug use, or any type of illicit drug use and positive testing for hepatitis B and HIV. Odds ratios for hepatitis C infection in obstetric patients with these risk factors ranged from 6.3 to 73. Conversely, positive testing for other

**Table 3** The prevalence of hepatitis C infection in patients with or without studied risk factors

| *Risk factor* | *% hep C in those 'with' risk factor* | *% hep C in those 'without' risk factor* | *Odds ratio* | *p-value* |
|---|---|---|---|---|
| Any drug use | 28 (5/18) | 3 (2/80) | 15 | 0.002 |
| IVDA | 57 (4/7) | 2 (1/55) | 73 | 0.001 |
| Nasal drug use | 33 (4/12) | 2 (1/50) | 25 | 0.004 |
| HIV | 57 (4/7) | 3 (3/95) | 41 | 0.001 |
| Hepatitis B | 15 (5/33) | 3 (2/73) | 6.3 | 0.029 |
| Gonorrhea | 0 (0/17) | 8 (7/89) | (0) | 0.59 |
| Chlamydia | 2 (1/59) | 13 (6/47) | 0.12 | 0.043 |
| Syphilis | 0 (0/4) | 7 (7/102) | (0) | 1.0 |

Note that chlamydia infection was associated with a lower risk of hepatitis C

STDs had either no association (gonorrhea, syphilis) or a negative association (chlamydia) with hepatitis C infection.

Multiple logistic regression analysis of the factors associated with hepatitis C infection confirmed only patient age ($p = 0.016$) and HIV-positivity ($p = 0.023$) as significant predictors of hepatitis C infection.

### DISCUSSION

This study found that STD-infected inner city obstetric patients who returned for hepatitis C screening had a relatively high prevalence of hepatitis C infection (6.6%). These patients, therefore, represent a high-risk group in whom screening for hepatitis C should have a significant yield. Hepatitis C testing was far more likely to identify occult infection than screening for syphilis.

Several interesting associations were identified in our analysis. Age was significantly associated with positive hepatitis C status. Increasing age has been found to be an independent risk factor for hepatitis C in a variety of populations[5–9]. Several previous studies of general obstetric clinic patients have also noted older women to be more likely to harbor hepatitis C[10–13]. It is currently unclear whether this association represents a cohort effect or merely that older women have an increasing number of lifetime opportunities to be exposed to this frequently undiagnosed, indolent disease through a variety of mechanisms. Regardless of the reason, it seems clear that women under the age of 25 are at lower risk for hepatitis C than women over the age of 25.

Hepatitis C was very strongly linked with HIV-positive status. Both diseases are spread efficiently through parenteral exposure and are both commonly seen in intravenous drug abusing populations. Previous work has suggested that pregnant women positive for both HIV and hepatitis C are more likely to transmit hepatitis C to their infants than HIV-negative, HCV-positive mothers[14,15]. A number of investigators have found that increasing viral titers of hepatitis C are associated with a greater risk of vertical transmission[15–18], Co-infection with HIV may allow higher hepatitis C viral titers through immunosuppression or there may be some more direct mechanism affecting hepatitis C virus infectivity[19]. Any pregnant woman infected with either HIV or hepatitis C should undergo testing for the other disease.

Univariate analysis revealed strong associations of hepatitis C with any illicit drug use, intravenous drug use and nasal drug use. These relationships failed to achieve statistical significance on multiple logistic regression analysis. This was probably due to the small numbers of hepatitis C-positive patients in our study and because most of the HIV-positive cases were also drug users. Many previous studies have found illicit drug use to be a strong risk factor for hepatitis C infection[4–7,11,17,20–31]. The method of acquisition of hepatitis C via intranasal drug use is unclear. It has been suggested[4] that epistaxis and shared devices might be a method of blood-borne exposure or alternatively that intranasal drug use is a surrogate marker for other high-risk behaviors that patients are unwilling to report. Of interest in this regard, one study which mailed anonymous questionnaires to newly diagnosed hepatitis C patients found that 99.8% (466/467) admitted to at least one parenteral risk factor[31]. Another paper found that almost half of their hepatitis C-positive pregnant women had not confessed a history of intravenous drug use to their midwife on intake, but did so only later when confronted with their positive status[32].

The association of hepatitis B with hepatitis C was much weaker, and disappeared completely on multivariate analysis. Hepatitis B can be spread through either sexual contact or parenteral exposure. The other STDs we examined (gonorrhea, chlamydia, syphilis) are almost entirely contracted through sexual contact with an infected partner. These three diseases all had a 'negative' correlation with hepatitis C, although this only reached statistical significance for women testing positive for chlamydia. Sexual transmission of hepatitis C seems to be inefficient. Previous studies have found that the majority of sexual partners of hepatitis C patients are not infected with hepatitis C[2,3,7,31,33–36]. One group of investigators[3] found 'no' cases of sexual transmission in couples co-habitating for less than 10 years. Hepatitis C virus has not been detected in the semen of infected men[37]. Two independent groups of investigators[35,38] both found that about 75% of sexual partners who tested positive for hepatitis C had their own parenteral risk factors. Together with this background information, our finding of the lack of an association between hepatitis C and those STDs which are transmitted sexually – not parenterally – in most cases (gonorrhea, chlamydia, syphilis) adds support to the concept that hepatitis C is 'not' transmitted efficiently through sexual contact.

Identification of hepatitis C infection in asymptomatic pregnant women is important for a number of reasons. One is the availability of promising new multi-drug treatment regimens which may significantly improve the long-term prognosis for patients with chronic active hepatitis C infection. A second reason is the risk of vertical transmission. Most authors have found few or no cases of infected children when their mothers had negative or low hepatitis C RNA titers, but significantly greater risks of an affected child in women with high RNA titers on quantitative PCR testing[14,16–18,39–43]. Infants infected with hepatitis C during pregnancy or childbirth have been reported to develop severe clinical hepatitis, jaundice or death[39]. Thus, the identification of pregnant women with hepatitis C (and possibly viral RNA titers) allows discussion with the patient about the risks of delivering a child with perinatal hepatitis C infection. The third reason is that the identification of asymptomatic hepatitis C infection allows for effective counseling. As endorsed by the CDC, a variety of measures designed to minimize other hepatic insults should be recommended to 'all' patients infected with hepatitis C: complete avoidance of alcohol, vaccination against hepatitis A and B and avoidance of medications or herbs with potential hepatotoxicity. Elimination or minimization of other hepatotoxic insults should ameliorate the effects of chronic hepatitis C infection and lead to less long-term morbidity and mortality.

One can develop two possible approaches for hepatitis C screening in obstetric patients. The first is to screen only high-risk groups. Studies from outside the US have usually reported that the pregnant population is not at higher risk for hepatitis C than the local population[10,13,32,39,44–46]. Most previous studies from within the US (which have looked at inner city clinic populations) have found that this group of obstetric patients are indeed at significantly greater risk for hepatitis C infection[11,12,47]. Reported prevalences have ranged from 3.6 to 5.2%, much higher than the general US population (1.8%). One study from Germany[48] also noted a higher risk for hepatitis C in clinic versus private patients. Our study attempted to use STD infection as a surrogate marker of high-risk behavior which would identify a group at increased risk for hepatitis C. (While we did identify a group at high risk for hepatitis C, we cannot be certain that this approach has actually worked until we ascertain the background prevalence in our population via a general screening program.) Careful analysis of our data has revealed that the elevated risk was mainly confined to older women, drug users and those who tested positive for HIV or hepatitis B. Younger women who did not abuse drugs, but tested positive for gonorrhea, chlamydia or syphilis were not at higher risk for hepatitis C infection. Based on our study then, one could limit hepatitis C screening to obstetric clinic patients aged 25 years or more, drug users or those testing positive for HIV or hepatitis B.

One can also make a good argument for universal screening of all pregnant women. The expected yield of 1.8% (general US population) is greater than the yield of other commonly recommended prenatal tests. The main argument against universal screening of pregnant women is that there are as yet no clear measures that have been proven to decrease the low (about 5%) but significant risk of vertical transmission. Although there seems to be less transmission when viral titers are low or undetectable, it remains to be proven that lowering those titers with medical therapy during pregnancy will reduce transmission. At the current time, there is inadequate literature to support any obstetric intervention to decrease vertical transmission. Studies examining the mode of delivery have had conflicting results, with some showing a lower risk of vertical spread with Cesarean delivery[14,18,43], while others noted no difference in transmission rates when Cesarean delivery was utilized[40,41]. Detection of viral RNA in children within 3 days of birth suggests that at least some cases of vertical transmission occur *in utero*[42]. Thus, it is not at all clear that performing an elective Cesarean delivery will reduce vertical transmission of hepatitis C virus.

Among STD-infected, inner city pregnant women, those aged 25 years or over, drug users or women with positive tests for HIV or hepatitis B are at high risk for hepatitis C infection and should be screened. Further study is needed to best define the at-risk population and to decide whether routine universal hepatitis C screening is warranted.

### REFERENCES

1. Centers for Disease Control and Prevention. Recommendations for prevention and control of hepatitis C virus (HCV) infection and HCV-related chronic disease. *MMWR* 1998;47:1–39
2. Chayama K, Kobayashi M, Tsubota A, *et al*. Molecular analysis of intraspousal transmission of hepatitis C virus. *J Hepatol* 1995;22:431–9
3. Akahane Y, Kojima M, Sugai Y, *et al*. Hepatitis C virus infection in spouses of patients with type C chronic liver disease. *Ann Intern Med* 1994;120: 748–52
4. Conry-Cantilena C, VanRaden M, Gibble J, *et al*. Routes of infection, viremia, and liver disease in blood donors found to have hepatitis C virus infection. *N Engl J Med* 1996;334:1691–6
5. Lapane KL, Jakiche AF, Sugano D, *et al*. Hepatitis C infection risk analysis: who should be screened? *Am J Gastroenterol* 1998;93:591–6
6. Hershow RC, Kalish LA, Sha B, *et al*. Hepatitis C virus infection in Chicago women with or at risk for HIV infection. *Sex Trans Dis* 1998;25: 527–32
7. Brusaferro S, Barbone F, Andrian P, *et al*. A study on the role of the family and other risk factors in HCV transmission. *Eur J Epidemiol* 1999;15: 125–32
8. Murphy EL, Bryzman S, Williams AE, *et al*. Demographic determinants of hepatitis C virus seroprevalence among blood donors. *JAMA* 1996; 275:995–1000
9. Guadagnino V, Stroffolini T, Rapicetta M, *et al*. Prevalence, risk factors, and genotype distribution of hepatitis C virus infection in the general population: a community-based survey in southern Italy. *Hepatology* 1997;26:1006–11
10. Alvarez-Munoz MT, Vazquez-Rosales JG, Torres-Lopez FJ, *et al*. Infection of pregnant women with hepatitis B and C viruses and risks for vertical transmission. *Arch Med Research* 1997; 28:415–9
11. Leikin EL, Reinus JF, Schmell E, Tejani N. Epidemiologic predictors of hepatitis C virus infection in pregnant women. *Obstet Gynecol* 1994;84:529–34
12. Magriples U, Bernstein P, Snyder E, Copel JA. Can risk factor screening predict hepatitis C antibody reactivity? *Am J Perinatol* 1998;15:395–8
13. Lima MPJS, Pedro RJ, Rocha MDC. Prevalence and risk factors for hepatitis C virus (HCV) infection among pregnant Brazilian women. *Int J Gynecol Obstet* 2000;70:319–26
14. Paccagnini S, Principi N, Massironi E, *et al*. Perinatal transmission and manifestation of hepatitis C virus infection in a high risk population. *Pediatr Infect Dis J* 1995;14:195–9
15. Zanetti AR, Tanzi E, Paccagnini S, *et al*. Mother-to-infant transmission of hepatitis C virus. *Lancet* 1995;345:289–91
16. Garland SM, Tabrizi S, Robinson P, *et al*. Hepatitis C – role of perinatal transmission. *Aust NZ J Obstet Gynaecol* 1998;38:424–7
17. Ohto H, Terazawa S, Sasaki N, *et al*. and the Vertical Transmission of Hepatitis C Virus Collaborative Study Group. Transmission of hepatitis C virus from mothers to infants. *N Engl J Med* 1994;330:744–750
18. Okamoto M, Nagata I, Murakami J, *et al*. Prospective reevaluation of risk factors in mother-to-child transmission of hepatitis C virus: high viral load, vaginal delivery, and negative anti-NS-4 antibody. *J Infect Dis* 2000;182:1511–4
19. Bonacini M, Puoti M. Hepatitis C in patients with human immunodeficiency virus infection. *Arch Int Med* 2000;160:3365–73
20. Flamm SL, Parker RA, Chopra S. Risk factors associated with chronic hepatitis C virus infection: limited frequency of an unidentified source of transmission. *Am J Gastroenterol* 1998;93:597–600
21. Delage G, Infante-Rivard C, Chiavetta JA, *et al*. Risk factors for acquisition of hepatitis C virus infection in blood donors: results of a case-control study. *Gastroenterology* 1999;116:893–9
22. Villano SA, Vlahov D, Nelson KE, *et al.* Incidence and risk factors for hepatitis C among injection drug users in Baltimore, Maryland. *J Clin Microbiol* 1997;35:3274–7
23. Garfein RS, Doherty MC, Monterroso ER, *et al.* Prevalence and incidence of hepatitis C virus infection among young adult injection drug users. *J Acq Immune Defic Synd Hum Retrovirol* 1998;18 Suppl 1:S11–19
24. Alter MJ, Kruszon-Moran D, Nainan OV, *et al.* The prevalence of hepatitis C virus infection in the United States, 1988 through 1994. *N Engl J Med* 1999;341:556–62
25. Butler TG, Dolan KA, Ferson MJ, *et al.* Hepatitis B and C in New South Wales prisons: prevalence and risk factors. *Med J Aust* 1997;166:127–30
26. Dubois F, Desenclos JC, Mariotte N, Goudeau A, and the Collaborative Study Group. Hepatitis C in a French population-based survey, 1994: sero-prevalence, frequency of viremia, genotype distribution, and risk factors. *Hepatology* 1997;25: 1490–6
27. Lamden KH, Kennedy N, Beeching NJ, *et al.* Hepatitis B and hepatitis C virus infections: risk factors among drug users in northwest England. *J Infection* 1998;37:260–9
28. Oliveira MLA, Bastos FI, Telles PR, *et al.* Prevalence and risk factors for HBV, HCV and HDV infections among injecting drug users from Rio de Janeiro, Brazil. *Braz J Med Biol Res* 1999;32:1107–14
29. Salleras L, Bruguera M, Vidal J, *et al.* Importance of sexual transmission of hepatitis C virus infection in seropositive pregnant women: a case-control study. *J Med Virol* 1997;52:164–7
30. Santana-Rodriguez OE, Male-Gil ML, Hernandez-Santana JF, *et al.* Prevalence of serologic markers of HBV, HDV, HCV and HIV in non-injection drug users compared to injection drug users in Gran Canaria, Spain. *Eur J Epidemiol* 1998;14:555–61
31. Sladden TJ, Hickey AR, Dunn TM, Beard JR. Hepatitis C transmission on the north coast of New South Wales: explaining the unexplained. *Med J Aust* 1997;166:290–3
32. Ward C, Tudor-Williams G, Cotzias T, *et al.* Prevalence of hepatitis C among pregnant women attending an inner London obstetrical department: uptake and acceptability of named antenatal testing. *Gut* 2000;47:277–80
33. Everhart JE, Di Bisceglie AM, Murray LM, *et al.* Risk for non-A, non-B (type C) hepatitis through sexual or household contact with chronic carriers. *Ann Int Med* 1990;112:544–5
34. Setoguchi Y, Kajihara S, Hara T, *et al.* Analysis of nucleotide sequences of hepatitis C virus isolates from husband–wife pairs. *J Gastroenterol Hepatol* 1994;9:468–71
35. Sun CA, Chen HC, Lu CF, *et al.* Transmission of hepatitis C virus in Taiwan: prevalence and risk factors based on a nationwide survey. *J Med Virol* 1999;59:290–6
36. Bresters D, Mauser-Bunschoten EP, Reesink HW, *et al.* Sexual transmission of hepatitis C virus. *Lancet* 1993;342:210–11
37. Semprini AE, Persico T, Thiers V, *et al.* Absence of hepatitis C virus and detection of hepatitis G virus/GB virus C RNA sequences in the semen of infected men. *J Infect Dis* 1998;177:848–54
38. Zylberberg H, Thiers V, Lagorce D, *et al.* Epidemiological and virological analysis of couples infected with hepatitis C virus. *Gut* 1999; 45:112–16
39. Kumar RM, Frossad PM, Hughes PF. Sero-prevalence and mother-to-infant transmission of hepatitis C in asymptomatic Egyptian women. *Eur J Obstet Gynecol Reprod Biol* 1997; 75:177–82
40. Fischler B, Lindh G, Lindgren S, *et al.* Vertical transmission of hepatitis C virus infection. *Scand J Infect Dis* 1996;28:353–6
41. Resti M, Azzari C, Mannelli F, *et al.* and the Tuscany Study Group on Hepatitis C Virus Infection in Children. Mother to child transmission of hepatitis C virus: prospective study of risk factors and timing of infection in children born to women seronegative for HIV-1. *BMJ* 1998;317:437–40
42. La Torre A, Biadaioli R, Capobianco T, *et al.* Vertical transmission of HCV. *Acta Obstet Gynecol Scand* 1998;77:889–92
43. Granovsky MO, Minkoff HL, Tess BH, *et al.* Hepatitis C virus infection in the mothers and infants cohort study. *Pediatrics* 1998;102:355–9
44. Floreani A, Paternoster D, Zappala F, *et al.* Hepatitis C virus infection in pregnancy. *Br J Obstet Gynaecol* 1996;103:325–9
45. Tanzi M, Belleli E, Benaglia G, *et al.* The prevalence of HCV infection in a cohort of pregnant women, the related risk factors and the possibility of vertical transmission. *Eur J Epidemiol* 1997;13:517–21

46. Moriya T, Sasaki F, Mizui M, *et al*. Transmission of hepatitis C virus from mothers to infants: its frequency and risk factors revisited. *Biomed Pharmacother* 1995;49:59–64
47. Silverman NS, Jenkin BK, Wu C, *et al*. Hepatitis C virus in pregnancy: seroprevalence and risk factors for infection. *Am J Obstet Gynecol* 1993; 169:583–7
48. Hillemanns P, Dannecker C, Kimmig R, Hasbargen U. Obstetrical risks and vertical transmission of hepatitis C virus infection in pregnancy. *Acta Obstet Gynecol Scand* 2000;79:543–7

RECEIVED 09/15/02; ACCEPTED 08/25/03
